# Epigenetics and Immunometabolism in Diabetes and Aging

**DOI:** 10.1089/ars.2017.7299

**Published:** 2018-07-20

**Authors:** Tomasz J. Guzik, Francesco Cosentino

**Affiliations:** ^1^ BHF Centre for Research Excellence, Institute of Cardiovascular and Medical Research (ICAMS), University of Glasgow, Glasgow, United Kingdom.; ^2^ Department of Internal and Agricultural Medicine, Laboratory of Translational Medicine, Jagiellonian University Collegium Medicum, Krakow, Poland.; ^3^ Cardiology Unit, Department of Medicine, Karolinska Institute, Karolinska University Hospital, Stockholm, Sweden.

**Keywords:** vascular, inflammation, epigenetics, nitric oxide, superoxide, diabetes

## Abstract

***Significance:*** A strong relationship between hyperglycemia, impaired insulin pathway, and cardiovascular disease in type 2 diabetes (T2D) is linked to oxidative stress and inflammation. Immunometabolic pathways link these pathogenic processes and pose important potential therapeutic targets.

***Recent Advances:*** The link between immunity and metabolism is bidirectional and includes the role of inflammation in the pathogenesis of metabolic disorders such as T2D, obesity, metabolic syndrome, and hypertension and the role of metabolic factors in regulation of immune cell functions. Low-grade inflammation, oxidative stress, balance between superoxide and nitric oxide, and the infiltration of macrophages, T cells, and B cells in insulin-sensitive tissues lead to metabolic impairment and accelerated aging.

***Critical Issues:*** Inflammatory infiltrate and altered immune cell phenotype precede development of metabolic disorders. Inflammatory changes are tightly linked to alterations in metabolic status and energy expenditure and are controlled by epigenetic mechanisms.

***Future Directions:*** A better comprehension of these mechanistic insights is of utmost importance to identify novel molecular targets. In this study, we describe a complex scenario of epigenetic changes and immunometabolism linking to diabetes and aging-associated vascular disease. *Antioxid. Redox Signal.* 29, 257–274.

## Introduction

The prevalence of obesity and type 2 diabetes (T2D) mellitus is alarmingly increasing worldwide (12, 71). The International Diabetes Federation currently estimates that 415 million people have been diagnosed with diabetes mellitus worldwide and anticipate an increase up to 640 million by the year 2040 (1). The main determinants of this increase are represented by modifiable (sedentary lifestyle and dietary habits) and nonmodifiable factors (genetic predisposition and aging; 119). T2D is associated with increased risk of micro- and macrovascular complications and approximately twofold greater mortality when compared with the general population (71). Advances in therapy have reduced T2D morbidity and mortality. However, cardiovascular risk is far to be eradicated, and mechanism-based therapeutic approaches are needed (42). In patients with T2D, high glucose levels trigger endothelial inflammation, mitochondrial oxidative stress, and reduced availability of nitric oxide (NO), all contributing to cardiovascular complications. One of the key predictive factors related to micro- and macroangiopathy is associated with accelerated vascular aging resulting in atherosclerosis and microvascular dysfunction (9, 144). Low-grade inflammation has been established as one of the key mechanisms linking these conditions (51, 97, 98, 113, 138). Therefore, T2D is a prime example of an interplay between metabolism and immunity, making it prototypic for an in-depth look into immunometabolism. It has been known since the 1980s that insulin and insulin receptors modulate immunity (64). At the same time, low-grade inflammation and the infiltration of immune cells into insulin-sensitive tissues lead to metabolic impairment and accelerated aging (145). Perivascular and adipose tissue (AT) inflammatory infiltrate, and altered immune cell phenotype, can precede the development of metabolic disorders, including obesity, insulin resistance, T2D, atherosclerosis (129, 150), or hypertension (102). Moreover, specific ablation of macrophages, B cells, or T cells from the AT can not only restore metabolic function but also prevent development of related pathologies (51, 145). Clinical significance is emphasized by the prognostic value of C-reactive protein levels or plasma cytokines such as interleukin (IL)-6 or tumor necrosis factor alpha (TNF-α; 74), although ongoing clinical trials will give us strong insight soon. Better understanding of immunometabolic diseases may lead to the development of novel immune targeted therapies in the treatment and prevention of metabolic dysfunction in hypertension, diabetes, and aging. Epigenetic mechanisms that control immune cell lineage determination, function, and migration are implicated (132, 149) and can provide valuable therapeutic targets in the future.

Epigenetic modifications are emerging as key players in the setting of this pathogenetic chain of events (61). Acetylation and methylation at DNA/histone complexes significantly alter gene expression by modulating chromatin accessibility (21).

## Accelerated Vascular Aging in Diabetes

**FIG. 1.**
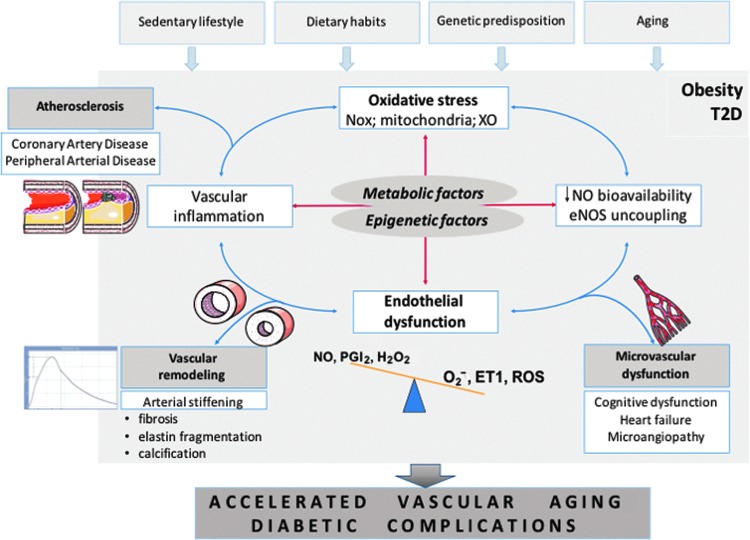
**Vicious cycle of oxidative stress, endothelial dysfunction, and vascular inflammation in the pathogenesis of vascular complications of metabolic disorders.** eNOS, endothelial nitric oxide synthase; ET1, endothelin 1; H_2_O_2_, hydrogen peroxide; NO, nitric oxide; Nox, nonphagocytic NADPH oxidase; O_2_^−^, superoxide anion; PGI_2_, prostacyclin; ROS, reactive oxygen species; T2D, type 2 diabetes; XO, xanthine oxidase.

Accelerated vascular aging is characterized by progressive pathological vascular remodeling, dependent on vascular fibrosis and calcification, leading to vascular stiffening as a clinical manifestation and is particularly prevalent in T2D (Fig. 1) (172). It bears important prognostic significance (40). Extracellular matrix remodeling is initiated by risk factors such as hypertension and diabetes and is mediated by endothelial dysfunction and vascular inflammation (28). Factors affecting collagen deposition and matrix degradation are linked to pathologic vascular remodeling also in the context of inflammation (25, 92). MMP9 (metalloproteinase 9) is, for example, essential for driving macrophage-dependent inflammation in the context of aging (92), although the cause–effect relationship between matrix remodeling factors and cardiovascular outcomes remains poorly defined (31), and may indicate other important regulators. Telomere shortening is one of the features of accelerated vascular aging. Indeed, vascular telomere length is lower in T2D patients (162). This is also important as a recent cross-sectional study demonstrated that telomere length is independently associated with subclinical atherosclerosis in subjects with T2D (152). However, accelerated telomere attrition was recently reported in circulating leukocytes, but not arteries, in T2D compared to control rats (156). This indicates the importance of immune senescence in diabetic vascular dysfunction/aging pathogenesis and that leukocytes may be primary targets of accelerated aging.

## Immunometabolism: Basic Concepts

The relationship between immunity and metabolism is bidirectional and includes (i) the role of inflammation in the pathogenesis of metabolic disorders, such as diabetes, obesity, metabolic syndrome, and hypertension and (ii) the role of metabolic factors in regulation of immune cell functions (132). The latter encompasses the effects of metabolic state of the environment on inflammation and the metabolic processes within the immune cells that regulate immunity (112).

It has been well recognized that metabolic state of environment may affect the development of inflammation (11, 87) particularly by affecting substrates available and also by changing chemokine gradients and local cytokine production. Overabundance of substrates observed in obesity and metabolic syndrome affects the phenotype of both infiltrating and resident immune cells (145). This has been strongly demonstrated in relation to macrophage phenotype switching between M1 and M2 (190).

### Macrophages

**FIG. 2.**
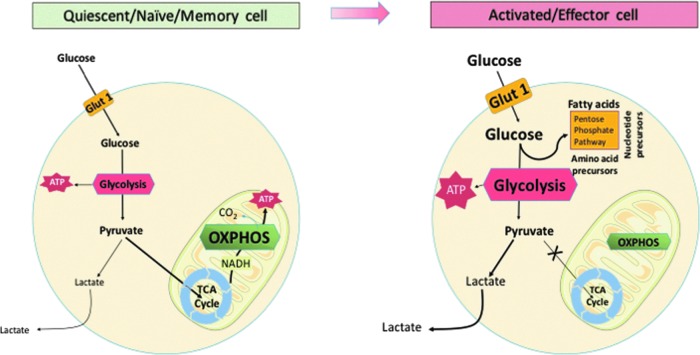
**Schematic representation of metabolic alterations between OXPHOS and anaerobic glycolysis is one of the key determinants of immune cell activation (*e.g.*, macrophage or T cell) from quiescent state.** Modified, based on (36) TCA/Krebs cycle; ATP; Modified from ATP, adenosine triphosphate; OXPHOS, oxidative phosphorylation; TCA, tricarboxylic acid.

More importantly, however, immune responses are accompanied by a dramatic metabolic switch within the immune cells themselves (145). For example, interferon gamma (IFN-γ)-activated (M1 type) macrophages rapidly shift to aerobic glycolysis, while M2-type macrophages rely on oxidative phosphorylation (Fig. 2). This has been first identified nearly five decades ago in studies of peritoneal macrophages demonstrating their increased glycolysis and decreased oxygen consumption on activation (62).

### T cells

Similar metabolic switches are related to adaptive T cell responses. Naive and quiescent T cells rely on glucose and fatty acid metabolism for energy, such as the tricarboxylic acid cycle, linked to the generation of adenosine triphosphate (ATP) *via* oxidative phosphorylation (Fig. 2; Table 1; 36, 125). When antigen is presented during immune challenge, T lymphocytes engage pathways of anabolic metabolism, switching to aerobic glycolysis (regulated greatly by mechanistic target of rapamycin or mTOR), to support clonal expansion and the development of effector functions (Fig. 2; Table 1; 107). T regulatory cells (Treg) are, in turn, dependent on oxidative phosphorylation and lipid peroxidation (93, 101). T cell activation is associated with transient activation of AMP-activated protein kinase (AMPK), a sensor of cellular energy levels, which allows the cells to prepare for high-energy consuming processes that follow T cell receptor activation (157).

**Table 1.** Major Immune Cell Populations Infiltrating Adipose Tissue, Their Role in Insulin Resistance, Key Effector Mechanisms, and Metabolic Regulation of Their Function

*Cell type*	*Effect on insulin resistance*	*Key effector mechanisms*	*Metabolic program*	*Key metabolic regulator*
Myeloid cells				
M1 Mf	⇑	TNF; IL-6; iNOS	Aerobic glycolysis	mTOR/HIF1f/Glut PFK2
M2 Mf	⇓	IL-10; arginase	Oxidative phosphorylation	AMPK; STAT6
Dendritic cells	⇑	IL-12; IL-15	Aerobic glycolysis (in activated state)	mTORC1; mTORC2
Mast cells	⇑	Histamine; PGE2; TNF	Aerobic glycolysis and oxidative phosphorylation	mTOR? AMPK
Neutrophils	⇑	MPO; IL-8; IL-1b; NETs	Glycolytic	mTORC1
Eosinophils	⇓	IL-10; IL-13; TGFb; IL-13	Glycolytic	AMPK
Lymphoid cells				
*Naive T cells*			Mixed fuel oxidative phosphorylation	
*Effector T cells*			Aerobic glycolysis	
T h cells (CD4+)				
Th1	⇑	IFN-γ; Tbx21	Aerobic glycolysis	mTORC1
Th2	⇓	IL-4; IL-5; IL-13	Aerobic glycolysis	mTORC1; mTORC2
Th17	⇑	IL-17	Aerobic glycolysis	mTORC1; HIF-1a
Treg (FOXP3+)	⇓	IL-10; TGFb	Lipid oxidation	AMPK
T c (CD8+)	⇑	TNF; IFN-γ (perforin/granzyme)	Aerobic glycolysis	mTORC1
*Memory T cells*			Lipid oxidation	TRAF6; AMPK
NK cells	⇑	TNFa; IFN-γ; IL4; IL13	Aerobic glycolysis	(mTORC1)
B cells	⇑	IgG	?	?

For detailed discussion and references see text (5, 37, 60, 64, 96, 105, 113, 131, 154, 166).

AMPK, AMP-activated protein kinase; IFN-γ, interferon gamma; IgG, immunoglobulin G; IL, interleukin; iNOS, inducible nitric oxide synthase; M1/M2, types of macrophages; Mf, macrophage; mTOR, mechanistic target of rapamycin; TNF, tumor necrosis factor; TRAF, TNF receptor-associated factor.

### Glucose metabolism and immune activation

The metabolic changes within the T cell during activation are modulated by environmental factors, such as insulin, which promotes T cell activation (64). Classical T cell activation is accompanied by upregulation of the insulin receptor, with subsequent increase in Glut1, Glut3, and Glut4, as well as an upregulation of glycolytic enzymes (37). These events are required for efficient adaptive immunity. Silencing the insulin receptor impairs T cell functions related to glucose transport and glycolysis, including polyclonal activation of CD4+ T cells, effector cytokine (Th1 type—IFN-γ and TNF and Th17 type—IL-17) production, migration, and proliferation (37). This was associated with alterations in intracellular signaling pathways, including RAS/ERK, PI3K/AKT, and mTOR pathways (37). The cytotoxicity of CD8+ T cells in response to alloantigens is also dependent on insulin receptor (37). Moreover, recent evidence suggests that regulatory T cells (Tregs) express the insulin receptor, and that high levels of insulin impair the ability of Tregs to suppress inflammatory responses *via* effects on the AKT/mTOR signaling pathway (60). The effect of insulin on Treg suppression is limited to IL-10 production and does not alter other suppression mechanisms.

Apart from the key role of mTOR in regulation of immune cell metabolism, particularly interesting data are related to the role of AMPK. AMPK is not only an important sensor of the cellular energy levels but through its potential inhibition by metformin may represent a potentially important pharmacological target for modulation of immunometabolism as well (5). Metformin, an activator of AMPK, inhibits Th1 and Th17 cell differentiation (76), while enhancing Treg through metabolic effects on fatty acid oxidation and glycolysis, leading to anti-inflammatory effects *in vivo* (151). This role of AMPK also provides a link between immunometabolism and oxidative stress.

### Therapeutic implications of immunometabolism

One of the key concepts of immunometabolism is related to the fact that immune cells can be reprogrammed by interfering with their metabolic states. This creates a possible therapeutic utility. M2 macrophage profile is promoted on inhibition of glycolysis (*e.g.*, by inhibiting pyruvate kinase M2; 116). Similarly, proinflammatory IL-17 producing T cells can be reprogrammed to develop into Treg-like cytokine producing profile by inhibition of glycolysis (*e.g.*, using 2-deoxyglucose).

## Immunometabolism of Diabetes

Accumulating evidence suggests that development of vascular complications of diabetes is dependent on interactions between immune cells and vascular wall components (70, 141). Indeed, immune cell infiltration is a key feature linking obesity to diabetes, as proinflammatory cytokines, macrophages, and T cells are essential for the development of insulin resistance (110). Both innate immunity and adaptive immunity contribute to metabolic pathology. A classical example is that the activation of toll-like receptors, IL-1 receptor type I or TNF receptor, results in nuclear factor kappa B (NF-κB) and Jun amino-terminal kinase signaling, leading to insulin receptor substrate (IRS)-1 and IRS-2 serine phosphorylation causing insulin resistance (112). Recognition of “metabolic” danger signals (such as glucose, ATP, or cholesterol) by the nucleotide oligomerization domain (NOD)-like receptor (NLR) family leads to activation of the NLR pyrin domain-containing 3 (NLRP3) inflammasome. This results in M1 macrophage activation (164). Abundance of fatty acids in obesity promotes AT inflammation in a toll-like receptor 4 (TLR4)-dependent manner (147). In healthy, nonobese individuals, Th2 and Treg residing in the fat have a beneficial effect by reducing visceral adipose tissue (VAT) inflammation. During obesity and other metabolic challenges, these cells are overwhelmed by proinflammatory CD8+ cells and Th1 CD4+ cells, which promote insulin resistance and glucose intolerance (174). CD4+ T cells have been recognized as a central regulator of chronic VAT inflammation, as they can modulate macrophage- and other T and B cell-dependent inflammatory responses. For example, IFN-γ-producing Th1 cells enhance proinflammatory macrophage activation in the AT, and IL-17 produced by Th17 cells may impair insulin receptor signaling in macrophages in culture and in surrounding AT (160). Th2 cells secreting IL-4 and IL-13 as well as the FOXP3+ Treg induce, in turn, anti-inflammatory macrophages that release IL-10, inhibiting low-grade inflammation in VAT (35, 105). Further characterization of AT infiltrating T cells in obesity revealed that they represent features of senescence-associated T cells typically seen in aging in the secondary immune organs. They promote chronic VAT inflammation and metabolic disorders by secreting large amounts of osteopontin (148). These CD153+PD-1+CD44hiCD4+ T cells are remarkably increased and preferentially accumulated in the VAT of high-fat diet-fed mice in a B cell-dependent manner. Indeed, B cells are critical for the pathogenesis of insulin resistance and metabolic dysfunction (173). Treatment with a clinically available anti-CD20 antibody, which results in significant reductions of B cells, attenuates disease. In contrast, transfer of immunoglobulins G (IgGs) from obese mice to controls leads to the development of insulin resistance (173). B cells worsen glucose tolerance, in part, by inducing proinflammatory cytokine production by T lymphocytes. Less evidence is available in the clinical setting, but insulin resistance in obese humans has been shown to be linked to elevated IgG autoantibodies (173) and immune cell accumulation in VAT, in particular, activated CD4+ and CD8+ T cells (177).

While immune cells can promote insulin resistance and T2D, hyperinsulinemia, as previouly discussed, alters immunity by promoting T cell activation as well as it increases T cell glucose uptake, amino-acid transport, and lipid metabolism (64). These changes promote overall decrease in IL-10 production with a parallel increase in production of IFN-γ, thus promoting a prodiabetic inflammatory milieu (60).

## Immunometabolic Determinants of Vascular Dysfunction in T2D

While AT inflammation is essential for the development of insulin resistance, diabetes is associated with perivascular adipose tissue (PVAT) inflammation, which leads to macrovascular and microvascular complications. We have recently shown that macrophage, T cell, and dendritic cell infiltration into PVAT precedes development of large vessel endothelial dysfunction and oxidative stress (129, 150). Molecular mechanisms of PVAT inflammation include signal transducer and activator transcription 4 (STAT4) in adipocytes and immune cells. STAT4 deficiency reduces development of atherosclerosis and PVAT inflammation in apolipoprotein E (ApoE)^−/−^ mouse (26) and in insulin-resistant obese Zucker rats (126). The immune dysfunction linking diabetes to vascular disease includes T effector cell memory polarization (6) and monocyte subset changes toward proinflammatory monocytes (103, 161, 171). PVAT inflammation is mediated by chemokines such as MCP-1 (monocyte chemoattractant protein 1), RANTES (regulated on activation, normal T cell expressed and secreted), or IP-10 (CXCL10) that recruit activated monocytes/macrophages and CD8+ T cells to PVAT (51, 54, 70, 113). Infiltrating cells release cytokines such as IFN-γ, TNF-α, or IL-6, which induce insulin resistance (19, 90, 91) and impair endothelium-dependent relaxation (82, 102, 103). IL-6 is also necessary for Th17 cell differentiation (14), another T cell subpopulation with strong proinflammatory impact on endothelial cells (ECs) and vascular smooth muscle cells (VSMCs) through activation of RhoA/Rho-kinase. It increases inhibitory endothelial nitric oxide synthase (eNOS) Thr495 phosphorylation in ECs leading to decreased NO production (108). Inflammatory cytokines modulate smooth muscle cell constriction, proliferation, and migration (99). They also affect adipokine release. For example, TNF, IL-6, and IL-17A can all inhibit expression of adiponectin or omentin-1, the vasoprotective adipokines (33, 69, 84, 170), and stimulate proinflammatory leptin and resistin (84, 111). Leptin, through its structural similarity to the cytokines of the long-chain helical family such as IL-6, IL-12, and IL-15, can affect leukocyte activation, chemotaxis, and release of oxygen radicals. In vascular cells, it induces proliferation of VSMCs and expression of adhesion molecules on ECs and VSMCs (84). These aspects have been reviewed by us in detail elsewhere (58, 113). It can also directly induce vascular dysfunction and oxidative stress (53, 57, 113) through possible effects on VSMC contractile function (176) and endothelial NO production.

## PVAT and Vascular Dysfunction

**FIG. 3.**
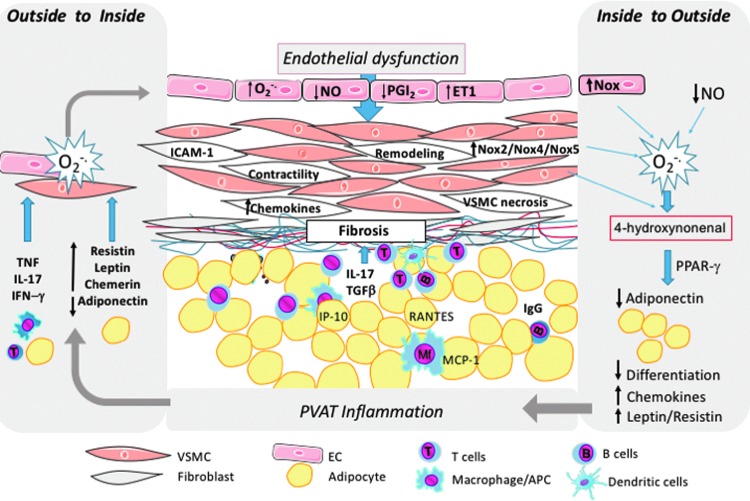
**Interactions between PVAT and vascular wall components “outside to inside” and “inside to outside” theory of interactions in development of vascular pathologies.** Both types of inteactions coexist in development of vascular dysfunction and augment each other. EC, endothelial cell; IFN-γ, interferon gamma; IgG, immunoglobulin G; IL, interleukin; PPAR-γ, peroxisome proliferator-activated receptor gamma; PVAT, perivascular adipose tissue; TNF-α, tumor necrosis factor alpha; VSMC, vascular smooth muscle cell.

While classically it is recognized that PVAT-derived adipokines and inflammatory cytokines affect EC and VSMC function, studies of human vascular dysfunction associated with metabolic impairment have led to a novel concept of an *inside-to-outside* signaling (Fig. 3; 7, 8, 96). According to this concept, bioactive compounds released from the vessel or the heart in conditions of increased oxidative stress can reciprocally control the biosynthetic activity of the neighboring perivascular or epicardial AT (7, 8, 96). While the mediators of this bidirectional cross talk are not clear, peroxidation products such as 4-hydroxynonenal that modulate gene expression within the PVAT or epicardial fat *via* PPAR-γ-dependent mechanisms have been shown (7, 8, 96). This may constitute an important mechanism through which endothelial dysfunction and oxidative stress can affect metabolism of surrounding AT. VSMCs also regulate PVAT inflammation by releasing chemotactic factors and contributing to immune cell recruitment and activation. Strong evidence implicates metabolic regulation of VSMC controlled inflammation (130). Indoleamine 2,3-dioxygenase, the first rate-limiting enzyme of the kynurenine pathway of tryptophan (Trp) degradation, has immune regulation and anti-inflammatory mechanisms in vascular inflammation and, mainly through effects on Treg function, regulates vascular cell adhesion molecule (VCAM)-1 expression and vascular recruitment of macrophages in mice. Such effect can be reversed by exogenous administration of the Trp metabolite 3-hydroxyanthranilic acid (130). Response of immune cells to VSMC-derived danger signals is also tightly regulated. For example, the innate immune protein CARD9 in macrophages may mediate necrotic smooth muscle cell-induced inflammation by activating NF-κB and contribute to neointima formation in vascular remodeling (89).

Finally, lymphatic vessel dysfunction is an emerging component of metabolic diseases (4). Lymphatics regulate tissue lipid accumulation, dyslipidemia, and edema. A recent study has demonstrated lymphatic dysfunction in diabetic db/db mice, which was rescued by L-arginine (140). These authors also demonstrated that PDE3 (phosphodiesterase 3) inhibition is required to maintain lymphatic vessel integrity and represents a viable therapeutic target for lymphatic endothelial dysfunction in metabolic disease (140).

Thus, over the years it became apparent that vascular dysfunction associated with diabetes is closely regulated by coincident immune and metabolic dysregulation making immunometabolic interventions a valuable therapeutic approach in the prevention and treatment of diabetic vascular disease.

## The Role of Epigenetics in Immunometabolic Regulation

**FIG. 4.**
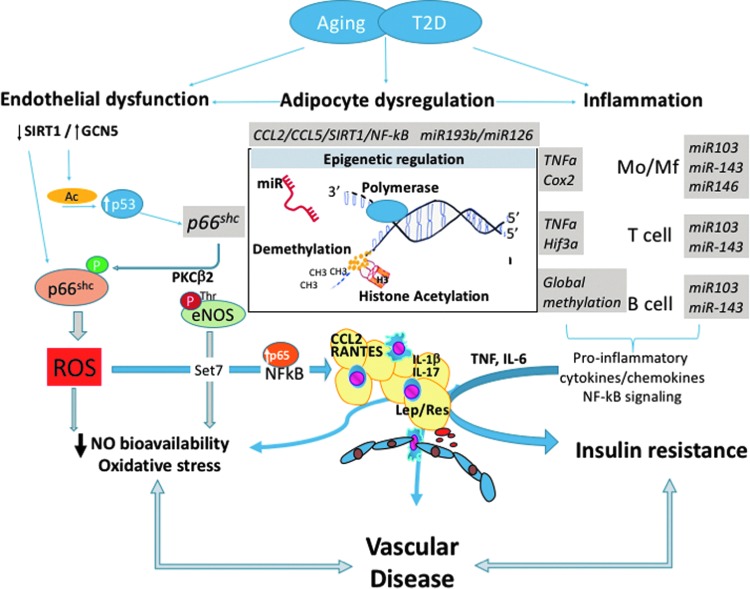
**Central role of epigenetic regulation in the pathogenesis of diabetic vascular dysfunction.** Epigenetic changes within endothelium, adipocytes, in particular PVAT and inflammatory cells are all contributing to vascular dysfunction and metabolic dysregulation, including insulin resistance. Key genes identified to be regulated epigenetically in each of the discussed organ systems are indicated in *gray* along with miRNAs implicated. These epigenetic changes lead to oxidative stress, adipocyte and perivascular inflammation, and endothelial dysfunction. CCL, CC chemokine ligand; miRNAs, microRNAs; NF-κB, nuclear factor kappa B.

The concept that adverse chromatin remodeling contributes to the pathogenesis of vascular damage in T2D has been introduced (78). Epigenetics is an important modulator of gene expression without affecting DNA sequence (26). Epigenetics leads to heritable changes in phenotype (61). The major mechanisms of epigenetic regulation are represented by DNA methylation, posttranslational histone modifications, and RNA regulating molecules such as noncoding RNAs (Fig. 4).

### Histone methylation and demethylation

DNA methylation can inhibit gene transcription through the covalent attachment of a methyl group to cytosine residues in CpG islands (79). In the setting of diabetes, promoter hypomethylation leads to increased expression of genes involved in inflammation, adiposity, β cell dysfunction, and vascular damage (88). Excessive free radical production is a major player for the onset of endothelial damage and impaired functionality. A better understanding of epigenetic changes affecting oxidant genes may unmask new mechanistic perspectives. Pathological chromatin remodeling causes gene expression changes that persist even after control of cardiovascular risk factors. Hypomethylation of the oxidant gene p66^Shc^ is contributing to the hyperglycemic memory in experimental diabetes (121). Indeed, high glucose-exposed ECs (human) and T2D mouse aortas show p66^Shc^ overexpression after restoration to normal glucose levels (121). p66^Shc^ upregulation and mitochondrial translocation induced free radical generation and impaired NO release. Global methylation status of leukocytes and B cells has been associated with insulin resistance and T2D (149, 187). Specific methylation changes were observed in TNF-α (65), ubiquitin-associated and SH3 domain-containing protein B (*UBASH3B*), or tripartite motif-containing 3 (*TRIM3*) genes involved in immune regulation (169). DNA methyltransferase DNMT3B is increased in macrophages exposed to high levels of saturated fatty acids, promoting M1 polarization in turn (181). Aberrant promoter DNA methylation also results in pathological endothelial-to-mesenchymal transition (EndMT) and subsequent fibrosis (179). Perivascular inflammation in turn is greatly orchestrated by RANTES receptor *CCR5* (CC chemokine 5 receptor) gene methylation (102).

### Histone acetylation and deacetylation

Histone acetylation mark was the first posttranslational modification identified (77) and the field has rapidly developed with the identification of enzymes that can either acetylate or deacetylate histones (21) and therefore lead to an opening of chromatin and subsequent transcription of relevant genes. On the contrary, nonacetylated histones are present in compact chromatin, also characterized by DNA hypermethylation at CpG. DNA and histone methyltransferase (DNMTs and HMTs), as well as histone acetyltransferase (HATs), are involved in plastic remodeling of chromatin as response to physiological and pathological stimuli (61). Together with histone deacetylases (HDACs) they regulate endothelial dysfunction and inflammation in T2D. One of the key families of deacetylases important in this setting are sirtuins (166), through the effects on vascular p66Shc gene transcription (SIRT1; 189). As a result, SIRT1 activation inhibits oxidative stress in the vessel wall and inhibits inflammation through prevention of NF-κB activation and cleavage of PARP—the poly (ADP-ribose) polymerase (188). SIRT1 is downregulated in the AT of obese individuals leading to histone hyperacetylation, which enhances macrophage recruitment, TNF, IL-6, IL-1β, TNF-α, IL-13, IL-10, and IL-4 expression, and generalized AT inflammation (43).

Through their effects on NF-κB activity, HATs and HDACs are important in controlling inflammation (166). HDAC3 regulates inflammatory genes in macrophages and HDAC2 contributes to resolution of inflammation (132). Role of SIRT1, HDAC4, enzyme involved in histone deacetylation is decreased in obesity and is correlated inversely to RANTES levels (2). T2D and T1D are both associated with increased H3 acetylation in the TNF-α and COX2 (cyclooxygenase 2) promoter regions, while H3 K4 methylation renders dysfunctional monocytes through effects on NF-κB-dependent genes (132). Methylation of lysine residue 9 of histone 3 in lymphocytes affects their autoreactive potential in type 1 diabetes (100) and suppressing the H3K9 methylation is proinflammatory in the vasculature (136, 167). A growing body of evidence suggests that the mammalian methyltransferase Set7, involved in methylation of histones, may represent an important mechanism of vascular damage under hyperglycemic conditions (32, 114, 153). In bovine and human ECs exposed to high glucose, Set7 induces monomethylation of lysine 4 of histone 3 (H3K4m1) on the promoter of the RelA gene encoding for the transcription factor NF-κB p65. This epigenetic modification by Set7 favors NF-κB p65 upregulation and resulting overexpression of adhesion molecules (32, 114, 153). Interestingly, suppression of Set7-dependent epigenetic changes prevented hyperglycemia-induced inflammation (32). Despite these data, the role of Set7 in patients with diabetes mellitus remained unknown. Thus, we designed a study to investigate the link between Set7-induced chromatin changes and vascular phenotype in patients with T2D. Our findings demonstrated that a specific epigenetic signature induced by Set7 regulates NF-κB p65 expression and, hence, contributes to dysregulation of oxidant/inflammatory genes and endothelial dysfunction (118).

Targeting this chromatin-modifying enzyme may represent a promising approach to maintain vascular homeostasis (186) and reduce oxidative and inflammatory burden in this setting.

### Noncoding RNAs

MicroRNAs (miRNAs) represent small noncoding RNAs that appear to play a key role regulating cardiovascular dysfunction in T2D (158). They posttranscriptionally regulate gene expression. Microarrays have demostrated a derangement of miRNA expression profile in patients with diabetes (185). Impairments of miRNAs involved in angiogenesis, inflammation, vascular repair, as well as endothelial homeostasis, have been reported (75, 104, 185). One of the hallmark studies has identified key miRNAs altered in subjects with T2D, as potential biomarkers. Lower plasma levels of miR-20b, miR-21, miR-24, miR-15a, miR-126, miR-191, miR-197, miR-223, miR-320, and miR-486 were seen in T2D and a modest increase of miR-28-3p. Importantly, reduced miR-15a, miR-29b, miR-126, miR-223, and elevated miR-28-3p levels antedated the manifestation of disease (185). Moreover, dysregulation of miRNAs within the AT, predominantly PVAT, has been linked to vascular disease, atherosclerosis, and aging (163). We recently investigated the miRNA landscape of the diabetic heart and its relationship with glycemic control (24). Our study was designed to address whether miRNAs may represent putative molecular drivers of hyperglycemic memory in the diabetic myocardium. miRNA landscape was assessed by miRNA polymerase chain reaction arrays in left ventricular specimens collected from streptozotocin-induced diabetic mice, with or without intensive glycemic control. We have shown that diabetes induces a profound alteration of miRNA expression in the heart and, most importantly, these detrimental signatures are not reverted by glycemic control (24). Such persistent alteration of several miRNAs orchestrating apoptosis, myocardial fibrosis, hypertrophy, autophagy, and redox signaling suggests the existence of hyperglycemic memory in the heart. Several miRNAs, which regulate inflammation, are decreased in T2D and aging, resulting in proinflammatory phenotype. MiR-21 has been widely associated with vascular aging and demonstrates concomitant effects on metabolic, inflammatory, and vascular mechanisms in the vessels and heart (Table 2; 29, 45, 180). While numerous other miRNAs have been implicated, miR-146b (resulting in monocyte activation), miR-107, (resulting in TLR4 expression and increased macrophage responses), miR-126 and miR-193b (resulting in enhanced chemotaxis) appear to be strongly linked to immunometabolism (132). Some proadipogenic and proinflammatory miRNAs are increased in T2D such as miR-103 and miR-143, resulting in increased adipocyte growth, altered adipokine profile, and insulin resistance (178), while miR-23b or novel miR-1298 is involved in VSMC phenotypic switching (72, 142). MiRNAs can also provide a molecular link between metabolic dysfunction and development of some of its complications such as hypertension (18, 95). Table 2 summarizes selected key miRNAs, which have been shown repetitively to regulate metabolic, immune, and vascular functions and diseases. Over the next few years, we will learn more about other types of noncoding RNAs in immune regulation, such as *lnc-DC*, which targets STAT3 or *Lethe*, which is induced by TNF and represses NF-κB target genes. These interesting new developments are reviewed elsewhere (80).

**Table 2.** Cross Talk Between Metabolic, Immune, and Vascular Functional Effects of Selected MicroRNAs Identified as Altered in Aging

*miR*	*Adipocyte/metabolic*	*Immune/inflammation*	*Vasculature*
miR-21	Adipogenic differentiation, apoptosis, PPARα downregulation; steatohepatitis	M1/M2 Mf balance, CD3+ T cells, Ly6c+cell content, T cell proliferation, FOXP3 expression	Angiogenesis, apoptosis, endothelial dysfunction, oxidative stress, proliferation
miR-124	Pancreatic islet development	Chemokine expression (CCL2); monocyte adhesion; Mf infiltration	Regulation of vascular function and response to hypoxia (through RhoA and Rac1).
miR-17/mir20a	Adipogenic differentiation, insulin resistance (GH/Ins/IGF-1); mitochondrial dysfunction	Monocyte differentiation; SIPRα expression Macrophage infiltration	Fibro-proliferative responses (through NOR-1)
let-7	Insulin resistance (GH/Ins/IGF-1); mitochondrial dysfunction; adipogenic differentiation	Inflammation; immune cell metabolism; inhibition of IκB leading to NF-κB activation	Angiogenesis; EC proliferation; BBB permeability
miR-27b	Adipogenic differentiation, adipocyte dysfunction; PPARγ expression	Regulation of NF-κB in RAW 264.9 cells; CXCL12 inhbition in adipocytes	Angiogenesis; HO-1 regulation; redox sensitive
miR-34a	Contributes to insulin resistance by targeting SIRT1; effects on aerobic glycolysis (*in silico*)	RCAN1 in ECs; repression of Wnt/β-catenin signaling	Endothelial dysfunction; mir34a is induced by p66^shc^; Induces VSMC senescence (SIRT-1)
miR-92	Adipogenic differentiation; cancer metabolism	Binding to CREB1, PTEN, and Bim in T cell regulation	Angiogenesis; vascular function; Nox4 regulation and H_2_O_2_ release; suppresses KLF4/KLF2 in ECs
miR-125b	Adipogenic differentiation, reduces IRF4; C/EBPa/PPARγ/FABP-4 and LDL expression	Stimulates TNF secretion; NF-κB activation; regulates mitochondrial and STAT3 metabolism in monocytes; reduces CCL4 in Mo and CD8+ (this lost in immunosenescence)	Endothelial dysfunction, oxidative stress
miR-130a	Represses adipogenesis and adipocyte differentiation (PPARγ);	Regulator of memory CD8+ T cell formation/immune senescence (through Tbx21)	Angiogenesis; vascular hypertrophy in hypertension; matrix remodeling; controls vasoconstriction in PAH (miR-130/301 family)
miR-29	Insulin resistance; upregulated following MR stimulation	regulator of memory CD8+ T cell formation	Vascular aging; osteoblastic VSMC differentiation; fibrosis; loss of elastin
miR-132	Insulin resistance; SIRT-1 suppression	MCP-1 and IL-8 release from AT and VSMC; NF-κB activation in Mf	Highly induced in VSMCs by Ang II; enhances CREB phosphorylation; vascular cell cycle, motility, and cardiovascular functions
miR-143	Adipogenic differentiation, adipocyte dysfunction, proliferation. Controls glycolysis by regulating hexokinase 2 (miR-155/miR-143)	Increase in sepsis; inhibits proinflammatory cytokines in T cells; increases IL-10; induced M2 Mf	Differentiation, proliferation; master regulators of EC and VSMC function; VSMC contractile differentiation
miR-155	Apoptosis, controls glycolysis by regulating hexokinase 2 (miR-155/miR-143); reduces obesity; NFLD; miR-155^−/−^ mice are a model of “obesity paradox” (178)	T cell activation; DC function; regulates Th17 induction; enhances T reg cells; stimulates proallergic responses	VSMC apoptosis; oxidative stress regulation; induces EC injury and atherogenesis
miR-145	Response to hypoxia; adipogenesis	Glycerol release and TNF in AT; activation of NF-κB; reduces expression of ADAM17	Upstream regulator of 20-HETE production in metabolic syndrome; role in PAH
miR-146b	Regulator of preadipocyte proliferation and differentiation; promotes adipogenesis (by SIRT1-FOXO1 suppression)	Innate immune transcriptome response; targets NF-κB signaling; induces TRAF6 gene and decreased IRAK1 in Mo.	Promotes VSMC proliferation and migration; suppresses EC hyperpermeability; represses EC activation; inhibits proinflammatory pathways in ECs

For details of original studies, please refer to specialized reviews discussing microRNAs in immune and metabolic diseases (25, 74, 110, 145, 148, 175).

Ang II, angiotensin II; AT, adipose tissue; BBB, blood/brain barrier; CCL, CC chemokine; CREB, cyclic AMP-response element-binding protein; CXCL, C-X-C motif chemokine; EC, endothelial cell; GH, growth hormone; HO-1, heme oxygenase 1; IGF-1, insulin growth factor 1; Ins, insulin; IRAK1, interleukin 1 receptor-associated kinase 1; KLF, kruppel-like factor; MCP-1, monocyte chemoattractant protein 1; miR, microRNA; Mo, monocytes; MR, mineralocorticoid receptor; NFLD, nonalcoholic fatty liver disease; NOR-1, neuron-derived orphan receptor-1; PAH, pulmonary arterial hypertension; PPAR, peroxisome proliferator-activated receptor; RCAN1, regulator of calcineurin 1; Tbx21, T helper cell type 1 transcription factor.

## Oxidative Stress, Vascular Inflammation, and Endothelial Insulin Resistance

Impaired insulin signaling and high glucose are strongly interlinked with cardiovascular disease (CVD) in the setting of T2D (12, 56, 59, 154). This relationship is strongly mediated by reactive oxygen species (ROS), through their effects on vascular inflammation and dysfunction (42, 55, 172). Insulin resistance precipitates the development of T2D and CVD (117). While links between hyperglycemia and oxidative stress are relatively clear, much less is known regarding the pathways through which free radicals regulate insulin resistance. Unquestionably, ROS contribute to altered insulin sensitivity in ECs. These effects may be, in part, direct and, in part, regulated by local low-grade inflammation promoted by oxidative stress. Vascular inflammation and atherosclerosis progression are directly linked to EC insulin signaling as demonstrated in ApoE^−/−^ mice (27, 135). Indeed, endothelial-specific overexpression of the inhibitory subunit of nuclear factor-kappa B (Iκ-Bα), which inhibits NF-κB activation, protects from insulin resistance in other organs (63). These findings cause a paradigm “shift” in the adipocentric theory (81). The novel concept that insulin resistance may primarily start in the endothelium is also strengthened by the fact that the endothelium lines the entire vascular system. Endothelial release of NO is essential for capillary recruitment and, hence, appropriate insulin delivery to hormone-sensitive organs (73). Accordingly, insulin-mediated glucose uptake is reduced in *eNOS^−/−^* compared with wild-type (WT) mice (10). Previous work has suggested that oxidative stress is a potent mediator of insulin resistance in ECs (27). Indeed, overexpression of free radical scavengers uncoupling protein 1 or manganese superoxide dismutase can restore endothelial NOS and prostacyclin (PGI_2_) synthase activities, thus warranting insulin-dependent vasodilation and anti-inflammatory effects (27, 57). Moreover, vasodilatation induced by insulin may importantly regulate insulin-mediated glucose uptake (20, 85, 133). Thus, restoration of endothelial function (measured as flow-mediated dilatation in human arteries) is clearly linked with an improvement of insulin sensitivity (106, 168). Key mechanistic markers of endothelial dysfunction and oxidative stress such as NF-κB activity or protein kinase C (PKC) β_2_ activity are elevated in the endothelium from patients with insulin resistance (50, 52, 155). In relation to this, we have recently studied the role of mitochondrial adaptor p66^Shc^ in ROS-driven insulin resistance in the ECs. p66^Shc^ silencing *in vivo* restored endothelial function through modulation of the IRS-1/AKT/eNOS (120). Knockdown of p66^Shc^ in endothelium from obese mice blunted free radical production and free fatty acid oxidation, key events favoring insulin resistance. Suppression of p66^Shc^-derived oxidative stress prevented dysregulation of NF-κB, advanced glycation end product (AGE) precursor methylglyoxal, and PGI_2_ synthase, biochemical effectors of maladaptive insulin signaling (120). In hypertension, angiotensin II infusion stimulates T cells to produce TNF, and etanercept (TNF-α antagonist) blunts vascular superoxide production (51). In macrophages, in turn, TNF-like weak inducer of apoptosis (TWEAK, Tnfsf12) and the receptor, fibroblast growth factor-inducible 14 (Fn14) promote ROS production and enhance nicotinamide adenine dinucleotide phosphate (NADPH) oxidase activity, which contributes to vascular damage and dysfunction in atherosclerosis (94).

A turn away from the sole “adipocentric” view of metabolic dysfunction origins is further supported by studies in mice with vascular smooth muscle-targeted deletion of p22phox subunit of the NADPH oxidase (183). p22phox is essential for activity of Nox1, Nox2, and possibly Nox4 NADPH oxidase, while Nox5 (not expressed in mice) is p22phox independent (47–50, 52, 143). These mice have significantly reduced vascular oxidative stress and are protected from endothelial dysfunction in a number of pathological conditions. Interestingly, high-fat feeding did not induce weight gain or leptin resistance in these mice, which was associated with strongly reduced T cell infiltration of perivascular fat. This is important as it indicates a causal immunometabolic link, suggesting that vascular dysfunction and inflammation may be primary, not secondary, in the development of obesity and insulin resistance (183). It also contributes to understanding of potential mechanisms of the inside-to-outside theory of the role of PVAT in vascular disease.

In T2D, increased glucose levels cause excessive free radical production from the mitochondria leading to the generation of AGEs, PKC activation, as well as increases in NF-κB (109). PKCβ_2_ isoform is associated with endothelial dysfunction through its effects on ROS (155). PKCβ_2_ elicits its deleterious effect through activation of mitochondrial and NADPH oxidases by regulating major components in ROS generation—namely p66^Shc^ and p47phox phosphorylation (83, 117). PKC inhibitor inhibits the NADPH oxidase activity (46, 55, 131). *p66^Shc^*^−/−^ mice are protected against hyperglycemia-induced endothelial dysfunction and oxidative stress (17) and p66^Shc^ expression is increased in lymphocytes and monocytes (PBMCs) from subjects with T2D. Moreover, p66^Shc^ expression is correlated with plasma ROS marker (isoprostanes; 115). PKCβ_2_, important in ROS generation, regulates of NF-κB signaling in response to high glucose by reducing its Iκ-Bα. This results in inflammatory activation of the ECs with increased VCAM-1 expression (83).

The role of NADPH oxidases in regulation of vascular inflammation in diabetes hypertension or atherosclerosis is well known, although recent studies have suggested additional important metabolic links. For example, Nox1-mediated increase in ROS induced by sphingosylphosphorylcholine leads to consequent enhancement of voltage-gated Ca^2+^ entry and thus vasoreactivity (146).

Although the understanding of the regulation of oxidant and inflammatory genes remains challenging, it is clearly emerging that targeting specific molecular machineries may represent an interesting therapeutic possibility to reduce CVD in the setting of metabolic disease.

## Pathways Linking Aging and Immunometabolism

An increased body of evidence shows a link between aging, CVD, and impaired metabolism. Not only aging impairs intracellular signaling triggering metabolic alterations but also metabolic conditions, such as obesity, diabetes, and insulin resistance, anticipate vascular and cardiac senescence. It is emerging that a dynamic interplay among p66^Shc^, NAD-dependent deacetylase (Sirtuin 1; SIRT1), NF-κB, forkhead transcription factor (FOXO), AMPK, and activator protein-1 (AP-1) transcription factor JunD underlines pathologic cardiovascular phenotypes in this setting. Recent studies have demonstrated that the adaptor p66^Shc^ is an important molecular effector that may explain how aging relates to metabolic and CVD. Adaptor protein p66^Shc^ is an important source of intracellular ROS (22). On the contrary, *p66^Shc^*^−/−^ mouse models exposed to oxidative stimuli showed diminished ROS generation (16, 44). Several years ago, we observed that aging-induced impairment of endothelium-dependent relaxation to acetylcholine was not present in *p66^Shc^*^−/−^ (38). Accordingly, NO availability was not reduced in aged *p66^Shc^*^−/−^ mice (38). Activation of p66^Shc^ is indeed involved in adipogenesis, insulin resistance, and diabetes-related cardiovascular complications (13, 17). More recently, as already mentioned, we demonstrated an upregulation of p66^Shc^ in obese mice and the involvement in endothelial insulin resistance (120). Gene expression of p66^Shc^ is increased in mononuclear cells obtained from patients with T2D and coronary artery disease (39, 115). Based on this background, it is possible to conclude that p66^Shc^ fosters ROS accumulation, derangement of mitochondrial function, insulin resistance, and diabetes. Mitochondrial dysfunction is characterized in diabetes (heart) by changes in mitochondrial structure and, mechanistically, complex I defect with oxidative stress results increased fatty acid oxidation (165). This effect is mediated by enhanced protein lysine acetylation (165).

SIRT-1, a member of the family of nicotinamide adenine dinucleotide-dependent proteins termed sirtuins, has recently emerged as an important regulator of cardiovascular aging and inflammation (127). SIRT-1 protects the heart against aging features (3). Aging-induced SIRT-1 downregulation leads to the translocation of NF-κB p65 to the nucleus and hence increased expression of inflammatory genes (182). Epigenetic changes, such as increased DNA methylation and noncoding RNAs, modulate expression of sirtuins (30, 139, 184). The maintenance of SIRT-1 homeostasis is crucial for the repression of pathways involved in arterial aging such as FOXO pathway (15). SIRT-1 also controls the release of protective factors such as recently identified Fgf21 in cardiac myocytes (128). Pharmacological inhibition of SIRT-1 protects against aging, impaired metabolic profiles, and cardiovascular complications (175). Among different compounds, resveratrol is an activator of SIRT-1. Resveratrol-increased SIRT-1 activity blunts the expression of oxidant and inflammatory genes by inducing epigenetic changes at the promoter level (34). Indeed, SIRT-1-induced histone deacetylation reduces the accessibility of transcription factors to chromatin, thereby blunting p66^Shc^ gene expression (23, 123). Downregulation of sirtuins in this setting favors transcription of FOXO-dependent genes leading to apoptosis, cell-cycle arrest, ROS generation, and impaired metabolism. NF-κB is a transcription factor expressed in mammalian cells (124). Its activation triggers inflammatory pathways in the heart and vessels. It was recently shown that silencing of endothelial NF-κB prolongs life span and improves endothelial insulin resistance in a mouse model of obesity. Selective endothelial overexpression of NF-κB inhibitory subunit was protective against insulin resistance in other tissues (63). Impaired insulin signaling is indeed an important hallmark linking metabolic disease with premature aging (134).

JunD, which is a member of the AP-1 transcription factor family, is emerging as a key factor protecting from the development of vascular oxidative stress. AP-1 is a hetero- or homodimeric complex made of proteins belonging to the c-Fos, c-Jun, ATF (activating transcription factor), and CREB (cyclic AMP-response element-binding protein) families (68). The cellular environment (infections, stress, cytokines, and growth factors) regulates gene expression *via* AP-1 (68). JunD regulates cell growth and survival, through affecting antioxidant gene expression (41). This results in the fact that *JunD*^−/−^ mice are characterized by premature aging, shortened life span, and increased cancer development (86, 117, 159). JunD overexpression decreased oxidative stress and blunted redox signaling resulting in diminished cellular apoptosis (41, 117). *JunD*^−/−^ murine embryonic fibroblasts showed downregulation of antioxidant enzymes and increased NADPH oxidase expression (41). We demonstrated the relevance of JunD for cardiovascular homeostasis (122). We observed an aging-induced decrease of JunD expression leading to an imbalance between pro-oxidant and antioxidant enzymes with increased ROS production. Indeed, young mice lacking JunD showed early impairment of redox signaling, mitochondrial derangement, and endothelial dysfunction (86). Furthermore, the vascular senescence observed in young *JunD*^−/−^ animals was similar to that observed in old WT mice. An adverse epigenetic remodeling occurring at the level of JunD promoter is responsible for such age-induced downregulation of JunD (86). This finding agrees with the notion that epigenetics affects the expression of genes involved in aging, dismetabolic profiles, and cardiovascular injury (159). In peripheral blood monocytes isolated from old compared to young healthy volunteers, JunD expression was reduced. In light of these findings, JunD can be considered as a promising target to prevent or delay age-induced CVD. Accordingly, disruption or upergulation of JunD expression promotes pressure-dependent cardiac apoptosis, hypertrophy, and angiogenesis (137) and blunt phenylephrine-mediated cardiomyocyte hypertrophy (66). In patients with severe heart failure, JunD protein expression is reduced (67). *JunD*^−/−^ mice show hyperinsulinemia, as a result of oxidative stress-induced pancreatic islet vascularization (86). Interestingly, the metabolic derangements found in *JunD*^−/−^ mice were rescued by treatment with antioxidants (86). These data clearly indicate that JunD is an important effector in the interaction among aging, metabolism, and CVD.

## Conclusion

Vascular endothelial dysfunction, oxidative stress, and low-grade inflammation are common features of metabolic diseases and are closely interlinked. Glucose metabolism affects immune phenotype and regulates oxidative stress generating enzymes affecting the development of all features of vascular dysfunction. Recent studies suggest that vascular dysfunction, endothelial insulin resistance, and vascular inflammation may precede and cause the development of insulin resistance, obesity, and T2D rather than being their mere consequence. This change from the classical “adipocentric” theory of metabolic vascular disease may have significant diagnostic and therapeutic implications.

Distinct epigenetic changes in vascular cells, adipocytes, and immune cells are frequently observed in obesity and T2D, and these are associated with phenotypic and functional alterations of these cells. Targeting these chromatin-modifying enzymes may represent a promising approach to reduce oxidative and inflammatory burden in the setting of diabetic vascular dysfunction.

## Abbreviations Used

AGEs: advanced glycation end products
AMPK: AMP-activated protein kinase
Ang II: angiotensin II
AP-1: activator protein-1
ApoE: apolipoprotein E
AT: adipose tissue
ATP: adenosine triphosphate
BBB: blood/brain barrier
CCL: CC chemokine ligand
CREB: cyclic AMP-response element-binding protein
CVD: cardiovascular disease
CXCL: C-X-C motif chemokine
EC: endothelial cell
eNOS: endothelial nitric oxide synthase
FOXO: forkhead transcription factor
GH: growth hormone
H_2_O_2_: hydrogen peroxide
HAT: histone acetyltransferase
HDAC: histone deacetylase
HO-1: heme oxygenase 1
IFN-γ: interferon gamma
IGF-1: insulin growth factor 1
IgG: immunoglobulin G
IL: interleukin
iNOS: inducible nitric oxide synthase
Ins: insulin
IRAK1: interleukin 1 receptor-associated kinase 1
IRS: insulin receptor substrate
Iκ-Bα: inhibitory subunit of nuclear factor-kappa B
KLF: kruppel-like factor
M1/M2: types of macrophages
MCP-1: monocyte chemoattractant protein 1
Mf: macrophage
miRNAs: microRNAs
Mo: monocytes
MR: mineralocorticoid receptor
mTOR: mechanistic target of rapamycin
NADPH: nicotinamide adenine dinucleotide phosphate
NFLD: nonalcoholic fatty liver disease
NF-κB: nuclear factor kappa B
NO: nitric oxide
NOR-1: neuron-derived orphan receptor-1
Nox: nonphagocytic NADPH oxidase
OXPHOS: oxidative phosphorylation
PAH: pulmonary arterial hypertension
PGI_2_: prostacyclin
PKC: protein kinase C
PPAR: peroxisome proliferator-activated receptor
PVAT: perivascular adipose tissue
RANTES: regulated on activation, normal T cell expressed and secreted
RCAN1: regulator of calcineurin 1
ROS: reactive oxygen species
STAT4: signal transducer and activator transcription 4
T2D: type 2 diabetes
Tbx21: T helper cell type 1 transcription factor
TCA: tricarboxylic acid
TLR4: toll-like receptor 4
TNFα: tumor necrosis factor alpha
TRAF: TNF receptor-associated factor
Treg: T regulatory cells
Trp: tryptophan
VAT: visceral adipose tissue
VCAM: vascular cell adhesion molecule
VSMC: vascular smooth muscle cell
WT: wild type
XO: xanthine oxidase
